# The clinical mutatome of core binding factor leukemia

**DOI:** 10.1038/s41375-019-0697-0

**Published:** 2020-01-02

**Authors:** Sabrina Opatz, Stefanos A. Bamopoulos, Klaus H. Metzeler, Tobias Herold, Bianka Ksienzyk, Kathrin Bräundl, Sebastian Tschuri, Sebastian Vosberg, Nikola P. Konstandin, Christine Wang, Luise Hartmann, Alexander Graf, Stefan Krebs, Helmut Blum, Stephanie Schneider, Christian Thiede, Jan Moritz Middeke, Friedrich Stölzel, Christoph Röllig, Johannes Schetelig, Gerhard Ehninger, Alwin Krämer, Jan Braess, Dennis Görlich, Maria Cristina Sauerland, Wolfgang E. Berdel, Bernhard J. Wörmann, Wolfgang Hiddemann, Karsten Spiekermann, Stefan K. Bohlander, Philipp A. Greif

**Affiliations:** 1 Laboratory for Leukemia Diagnostics, Department of Medicine III, University Hospital, LMU Munich, Munich, Germany; 2 Experimental Leukemia & Lymphoma Research, Department of Medicine III, University Hospital, LMU Munich, Munich, Germany; 3 German Cancer Consortium (DKTK), Heidelberg, Germany; 4 German Cancer Research Center (DKFZ), Heidelberg, Germany; 5 Laboratory for Functional Genome Analysis at the Gene Center, LMU Munich, Munich, Germany; 6 Institute of Human Genetics, University Hospital, LMU Munich, Munich, Germany; 7 Department of Internal Medicine 1, University Hospital Carl Gustav Carus, Dresden, Germany; 8 Oncology and Hematology, St. John of God Hospital, Regensburg, Germany; 9 Institute of Biostatistics and Clinical Research, University of Münster, Münster, Germany; 10 Department of Medicine A, Hematology, Oncology and Pneumology, University of Münster, Münster, Germany; 11 Department of Hematology, Oncology and Tumor Immunology, Charité University Medicine, Campus Virchow, Berlin, Germany; 12 Department of Molecular Medicine and Pathology, Faculty of Medical and Health Sciences, University of Auckland, Auckland, New Zealand

**Keywords:** Acute myeloid leukaemia, Cancer genetics

## Abstract

The fusion genes *CBFB*/*MYH11* and *RUNX1*/*RUNX1T1* block differentiation through disruption of the core binding factor (CBF) complex and are found in 10–15% of adult de novo acute myeloid leukemia (AML) cases. This AML subtype is associated with a favorable prognosis; however, nearly half of CBF-rearranged patients cannot be cured with chemotherapy. This divergent outcome might be due to additional mutations, whose spectrum and prognostic relevance remains hardly defined. Here, we identify nonsilent mutations, which may collaborate with CBF-rearrangements during leukemogenesis by targeted sequencing of 129 genes in 292 adult CBF leukemia patients, and thus provide a comprehensive overview of the mutational spectrum (‘mutatome’) in CBF leukemia. Thereby, we detected fundamental differences between *CBFB/MYH11*- and *RUNX1/RUNX1T1*-rearranged patients with *ASXL2*, *JAK2, JAK3, RAD21*, *TET2,* and *ZBTB7A* being strongly correlated with the latter subgroup. We found prognostic relevance of mutations in genes previously known to be AML-associated such as *KIT*, *SMC1A,* and *DHX15* and identified novel, recurrent mutations in *NFE2* (3%), *MN1* (4%), *HERC1* (3%), and *ZFHX4* (5%). Furthermore, age >60 years, nonprimary AML and loss of the Y-chromosomes are important predictors of survival. These findings are important for refinement of treatment stratification and development of targeted therapy approaches in CBF leukemia.

## Introduction

The core binding factor (CBF) protein complex, composed of RUNX1 and CBFB, is important for myeloid differentiation and recurrently altered in acute myeloid leukemia (AML) through genomic rearrangements. The fusion gene *CBFB/MYH11* results from a pericentric inversion of chromosome 16, inv(16)(p13.1q22), or less commonly from a t(16;16)(p13.1;q22). A translocation between chromosomes 8 and 21 [t(8;21)(q22;q22)] underlies the *RUNX1*/*RUNX1T1* fusion. Both rearrangements disrupt CBF function, leading to a block of myeloid differentiation and ultimately to leukemia. The resulting so-called CBF leukemias constitute 10–15% of adult de novo AML cases and are associated with favorable prognosis [1, 2].

Mutations in the receptor tyrosine kinase genes *KIT* and *FLT3* and the proto-oncogenes *NRAS* and *KRAS* have been observed in up to 80% of CBF leukemia patients and probably serve as cooperating factors during leukemogenesis promoting proliferation [3–6]. Identification of these additional genetic abnormalities could be helpful in predicting treatment outcome [5, 7, 8], since 45% of CBF leukemia patients relapse after standard chemotherapy [9, 10].

To systematically identify those cooperating mutations, we performed whole-exome sequencing (WES) of 12 adult AML patients with CBF rearrangement.

Even though both fusion products are sharing a similar pathogenic mechanism, there is a considerable clinical, cytomorphological and molecular variability within CBF leukemia [8–11]. Hence, the question arises of whether patients with t(8;21) and inv(16) leukemia should be considered distinct entities. To answer this question on the basis of the underlying spectrum of molecular differences, we compared the mutation profile (i.e. ‘mutatome’) of 162 patients with inv(16) leukemia to 130 patients with *t*(8;21) leukemia by targeted sequencing of 129 genes. Furthermore, we correlated distinct molecular and chromosomal aberrations with clinical outcome.

## Materials and methods

### Patient samples

For exome sequencing, diagnostic bone marrow (BM) samples were collected from 12 patients diagnosed with CBF Leukemia according to standard French–American–British and World Health Organization criteria between 2003 and 2011. The patients were selected based on availability of suitable samples. The inv(16)(p13.1q22) [*n* = 8] or t(16;16)(p13.1;q22) [*n* = 2] was detected by standard chromosome banding analysis and fluorescence in situ hybridization. The fusion transcripts *CBFB/MYH11* type A (*n* = 8) and type D (*n* = 2) were confirmed by reverse-transcriptase polymerase chain reaction (RT-PCR). After intensive induction therapy, based on cytarabine and anthracyclines, complete remission (CR) was achieved in all patients (<5% BM blasts; *CBFB/MYH11* transcripts no longer detectable by RT-PCR). A matched BM sample at CR was used as normal control for exome sequencing. Exome sequencing of two patients with AML *t*(8;21) was performed as described in a previous study conducted in our laboratory [12].

For targeted gene sequencing, we used BM (*n* = 259) or peripheral blood (PB; *n* = 33) samples from 162 adult patients with newly diagnosed and untreated AML with *CBFB*/*MYH11*-rearrangement and from 130 patients with *RUNX1*/*RUNX1T1*-rearrangement. These 292 patients included the 12 cases initially analyzed by exome sequencing. The patient characteristics are provided in Table 1. If available, matched BM aspirates obtained during CR were analyzed to evaluate the somatic status of detected variants (*n* = 110, 38%). Patients were treated according to protocols of the AML Cooperative Group or Study Alliance Leukemia. Study protocols were approved by the ethics committees of the participating centers. Written informed consent for scientific use of surplus samples was obtained in accordance with the Declaration of Helsinki.

**Table 1:** Patient characteristics

CBF leukemia	*n* = 292
Outcome	
CR	96%
Relapse	45%
Death	33%
*CBFB/MYH11*	*n* = 162
inv(16)	131 (81%)
t(16;16)	16 (10%)
*CBFB*/*MYH11*, not specified	15 (9%)
Type A fusion	90 (83%)
Nontype A fusion	13 (12%)
Type D	7 (6%)
Type E	4 (4%)
Type S	2 (2%)
Fusion not specified	53 (33%)
de novo AML	147 (91%)
t-AML	13 (8%)
s-AML	2 (1%)
Blasts in BM, median (range)	62% (10–95%)
Blasts in PB, median (range)	47% (6–93%)
Age in years, median (range)	44 (17–83)
Male sex	54% (88/162)
Allogeneic HCT in 1st CR	12 (7%)
*RUNX1/RUNX1T1*	*n* = 130
de novo AML	114 (88%)
t-AML	12 (9%)
s-AML	4 (3%)
Blasts in BM, median (range)	55% (10–90%)
Blasts in PB, median (range)	44% (6–78%)
Age in years, median (range)	54 (16–79)
Male sex	60% (78/130)
Allogeneic HCT in 1st CR	12 (9%)

### Whole-exome sequencing

Genomic DNA was extracted from BM or PB samples, after enrichment of mononuclear cells (Ficoll), using QIAcube technology (Qiagen, Hilden, Germany). Three micrograms of genomic DNA from diagnostic as well as matched remission BM specimen was used for preparation of sequencing libraries as described previously [3]. Protein-coding sequences were captured using SureSelect human all exon 50 Mb kit version 3 (Agilent, Santa Clara, CA) according to the manufacturer’s instructions. Exome libraries were sequenced with 76-bp paired end reads on a Genome Analyzer IIx or HiSeq platform (Illumina, San Diego, CA). Sequence alignment and variant detection was performed as described previously [13].

### Custom-targeted sequencing

Candidate genes identified by exome sequencing (*n* = 47) and genes known to be recurrently mutated in AML (*n* = 82) were studied by targeted amplicon sequencing (HaloPlex®; Agilent Technologies, Santa Clara, CA) in 292 CBF leukemia patients in order to identify genes with recurring mutations. A custom gene-panel was designed using an online software (Sure Design, Agilent Technologies, Santa Clara, CA). Our panel comprises 129 genes and a total target sequence of 396.45 kbp (Supplementary Table S1). From 110 patients-matched CR samples were available and sequenced to exclude germline variants. Library preparation was performed according to the manufacturer’s protocol (HaloPlex^®^ target enrichment system for Illumina sequencing, Version D.4, March 2013) using 225 ng genomic DNA per patient sample. The resulting libraries were sequenced by performing 250-bp paired end reads on a MiSeq and HiSeq instrument (Illumina, San Diego, CA) to an average target coverage of 500×. Software and detailed parameters used for variant calling were previously described [14].

### Clinical outcome analysis

Statistical analyses were performed using R version 3.4.1 (R Foundation for Statistical Computing, Vienna, Austria). Only patients who received intensive treatment (*n* = 157 [*CBFB*/*MYH11*] and *n* = 127 [*RUNX1*/*RUNX1T1*]) were included in the clinical outcome analyses (median follow-up: 3.6 years). Twenty-four patients received an allogeneic stem cell transplantation (HSCT) in first CR. Since indications for HSCT in first CR were not uniform and HSCT significantly prolongs relapse-free survival (RFS) in our CBF cohort (Supplementary Fig. S1), survival analyses were censored at the timepoint of transplantation in first CR but not later during the course of the disease. Mutations with frequencies less than 5% in the examined groups and variables with missing values in more than 15% of the patients were excluded from the analysis.

Clinical outcome was visualized using the Kaplan–Meier method and log-rank tests were calculated without adjustment of *p* values. Correlations of inv(16) and t(8;21) mutations were assessed using the Mann–Whitney *U* test for measurement variables, the Pearson's chi-squared test for categorical variables and the Fisher’s exact test for binary variables. All tests were two tailed. In case of multiple testing, *p* values were adjusted using the Benjamini–Hochberg procedure. The false discovery rate cutoff was set at <0.05. Survival analysis was performed using Cox regression models. A multivariate Cox regression model was built using all variables with a *p* value < 0.1 in the univariate regression models. Hazard ratios (HR) with corresponding Wald-test *p* values and 95% confidence intervals (CI) are reported. Outcome parameters were defined according to recent European Leukemia Net recommendations [2]. All statistical tests were considered significant at *p* ≤ 0.05.

## Results

### Somatic mutation profile of 12 CBF leukemia patients

**Fig. 1:**
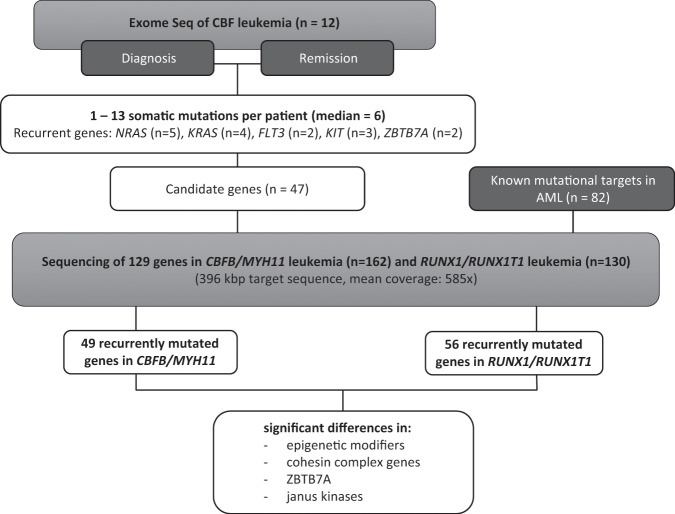
Study design. Exome sequencing of 12 adult patients with CBF leukemia was followed by custom-targeted sequencing of candidate genes from exome sequencing and known mutational targets in diagnostic samples of 162 patients with inv(16) and 130 patients with *t*(8;21). Ultimately, the mutation profiles were compared  between the two subgroups.

To systematically detect somatic mutations, we sequenced the protein-coding regions of 12 matched diagnostic and remission samples, generating at least 2.9 Gbp of raw sequence data from each exome (mean 5.1 Gbp). Thereby we covered more than 80% of RefSeq coding exon positions with a minimum read depth of 10 (Supplementary Table S2). The study design is outlined in Fig. 1. We identified 1–13 somatically acquired, leukemia-specific sequence variants per patient (median = 6). These include mutations in genes known to cooperate with CBF-rearrangements [e.g. *NRAS* (*n* = 6), *KRAS* (*n* = 4), *FLT3* (*n* = 2), *KIT* (*n* = 3), *ZBTB7A* (*n* = 2)] as well as in genes, which have not been described to be mutated in CBF leukemia so far (e.g. *ZFHX4, NFE2, HERC1*). Of note, targeted amplicon sequencing with higher coverage revealed several subclonal mutations of known AML driver genes (*FLT3*, *NRAS,* and *KRAS*), which were missed by exome sequencing. Results are summarized in Supplementary Fig. S2 and Table S3.

### Additional mutations in 162 *CBFB/MYH11*-rearranged AML samples

**Fig. 2:**
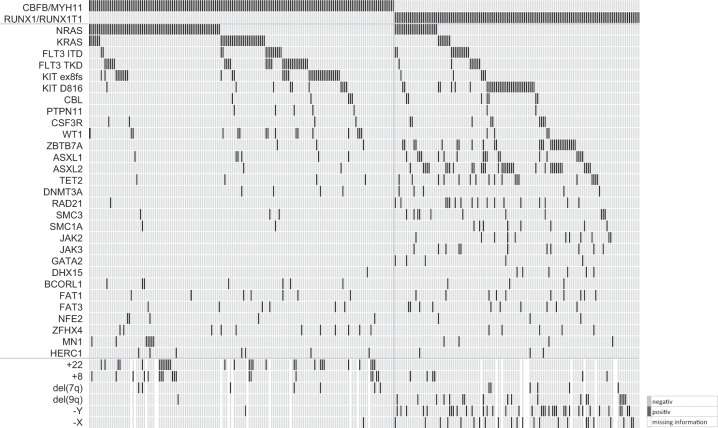
Results from targeted amplicon sequencing in 162 patients with *CBFB*/*MYH11*-rearrangement (left), and 130 patients with *RUNX1*/*RUNX1T1*-rearrangement (right). Each column represents one patient, each line shows the status of the indicated genetic aberrations.

By targeted sequencing, additional molecular mutations were detected in 156/162 patients (96%). Fifteen genes were found mutated in at least five patients (frequency ≥ 3,1%). Results are summarized in Fig. 2.

**Fig. 3:**
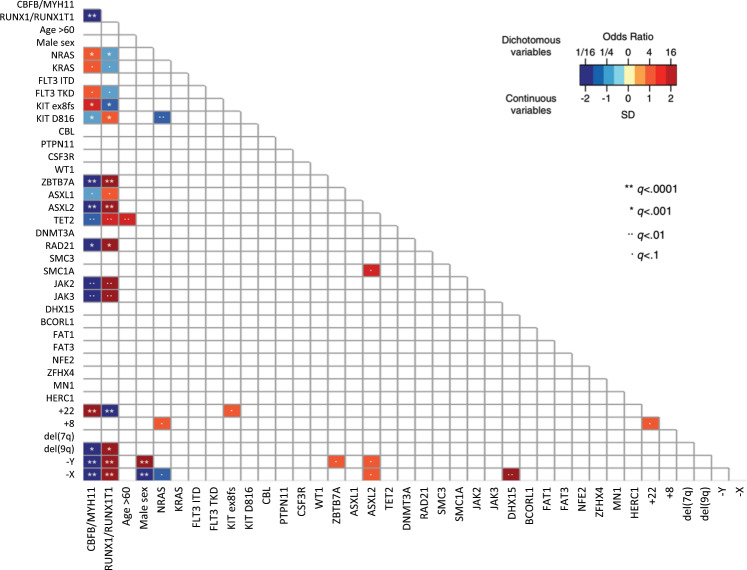
Correlation of inv(16) and *t*(8;21) leukemia with alterations common in CBF AML. Significance levels as followed: * = <0.05, ** = < 0.01, *** = < 0.001.

The mutation frequency of known CBF leukemia associated genes was similar to previous reports [3–6, 8]; Most common were missense mutations of *RAS*-genes, present in 57% of the patients (43% *NRAS*, 19% *KRAS*), affecting the known hotspots (G12, G13, Q61). *NRAS* mutations were highly associated with inv(16)-leukemia and trisomy of chromosome 8 but rarely found in patients with point mutations in *KIT* D816 (Fig. 3). Next in frequency were *FLT3* mutations in 27% of the cases. *FLT3-ITD* mutations were less common in our cohort than in cytogenetic normal AML (13/162; 8%), whereas point mutations or short deletions in *FLT3* (e.g. D835mut *n* = 18, N676K *n* = 8) had a frequency of 18% and were associated with the inv(16) subtype. A total of 26% of the *CBFB/MYH11*-positive patients had a *KIT* mutation, with 6% D816 point mutations (10/162) and 19% exon 8 frameshift mutations (31/162). Exon 8 frameshift mutations were highly associated with *CBFB/MYH11*-rearranged leukemia, while point mutations affecting position D816 were correlated with *RUNX1/RUNX1T1* leukemia (Fig. 3).

### Additional mutations in 130 *RUNX1/RUNX1T1*-rearranged AML samples

Signaling pathway alterations affecting *RAS*-genes and *FLT3* are less frequent in patients with *RUNX1/RUNX1T1*-compared with *CBFB/MYH11*-rearranged AML. There was no significant difference in the *KIT* mutation frequency, but while *KIT* exon 8 frameshift mutations are associated with *CBFB/MYH11*-rearranged leukemia (*n* = 31), they are underrepresented in *RUNX1/RUNX1T1* leukemia (*n* = 5) and here outnumbered by KIT D816 point mutations (*n* = 33 vs.10, 25%; Fig. 3). Mutations in janus kinases (*JAK*) were identified exclusively in patients with *RUNX1/RUNX1T1*-rearranged AML (16%). Consistent with recent reports [8, 12, 15–19], we found recurrent mutations in transcription factors [*ZBTB7A* (22%) and *GATA2* (4%)], chromatin modifiers [*ASXL1* (14%), *ASXL2* (29%), *EZH2* (2%) and *KDM6A* (2%)], genes involved in DNA methylation [*TET2* (14%), *DNMT3A* (4%), *IDH1* (2%) and *IDH2* (3%)], as well as mutations in genes that encode components of the cohesin complex [*RAD21* (14%), *SMC3* (8%), *SMC1A* (6%) and *STAG2* (2%)]. These common genetic changes are rare in inv(16) cases.

**Fig. 4:**
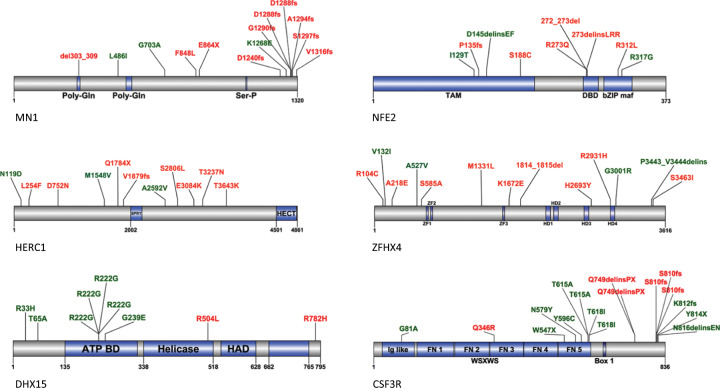
The location of mutations in the novel recurrently mutated genes *MN1*, *NFE2*, *HERC1*, *ZFHX4* and mutational hotspots in *CSF3R* and *DHX15*. Known protein domains are shown. Mutations colored in red were found in *CBFB/MYH11*-rearranged samples, mutations colored in green were found in our *RUNX1/RUNX1T1*-rearranged AML cohort.

In addition, we found recurrent mutations in the RNA helicase *DHX15* in 5% of patients (6/130) with a mutational hotspot in the ATP-binding domain (R222; Fig. 4). Sequencing results are shown in Fig. 2.

### Additional mutations shared in both CBF leukemia subtypes

Alterations of *CBL* were found in a total of 12 patients (4%) and *PTPN11* was found mutated in 7 patients (2%). In addition, we found recurrent *CSF3R* mutations in 13 patients (5%), with a hotspot in the extracellular fibronectin-like type VI domain (e.g. T615A and T618I) and mutations that truncate the cytoplasmic domain (e.g. S810fs). In addition to common signaling pathway mutations, we found 20 patients with *WT1* mutations causing a frameshift in exon 6 (7%) and 10 patients with alterations in *BCORL1* (3%).

Mutations in the FAT protocadherin family members, *FAT1* and *FAT3* were found in 17 and 16 patients, respectively (6%). In addition, we identified 16 patients with mutations in *ZFHX4* (6%), 11 patients with alterations in *MN1* (4%), 8 patients with *NFE2* (3%), and 10 patients with *HERC1* (3%) mutations. Of note, 3 out of 8 *NFE2* mutations are affecting position R273 in the DNA-binding domain (DBD), suggesting a mutational hotspot. Further, we identified hotspot mutations in *MN1* resulting in loss of the C-terminal part (Fig. 4).

Regarding the 33 PB samples, the mutational pattern is similar to the mutation profile of the BM samples (Supplementary Fig. S3). Range of the PB blast count was 6–93% (Median: 40%, Mean: 45.6%) and even in samples with low blast count, common mutations could be identified. This is likely due to the enrichment of mononuclear cells (including blasts) by Ficoll gradient during the sample preparation. Ultimately, the exact content of blasts in the samples analyzed by sequencing cannot be determined but is likely higher than in the initial samples evaluated by cytomorphology.

### Clinical relevance of recurring mutations

**Fig. 5:**
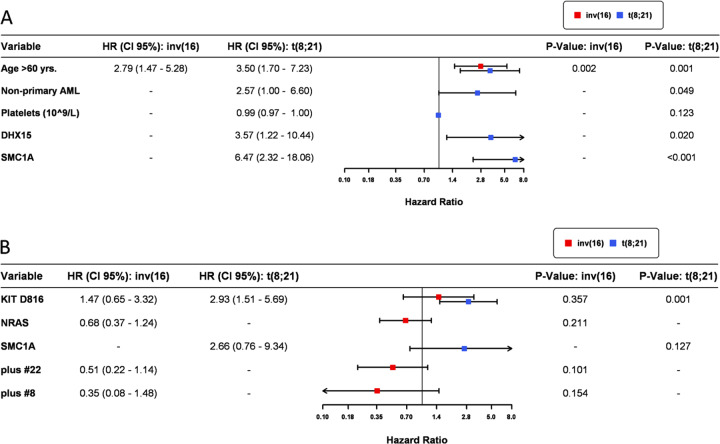
Impact of selected mutations on outcome in CBF leukemia. **a** overall survival and **b** relapse-free survival. Multivariate Cox regression. CBFB/MYH11-rearranged leukemia is depicted in red, RUNX1/RUNX1T1 in blue. OS was defined from the date of treatment initiation to the date of death. Relapse-free survival was calculated from the date of complete remission to the date of relapse or death in remission. Hazard ratios (HR), with corresponding Wald-test *p* values and 95% confidence intervals (CI) are reported. *p* values are considered significant at *p* ≤ 0.05.

There was no significant difference in OS (overall survival) or RFS between patients with *RUNX1*/*RUNX1T1*-rearranged AML or those with *CBFB*/*MYH11*-rearranged AML (Supplementary Fig. S4). Patients with CBF AML aged above 60 years had a shorter OS than younger patients (HR = 2.79 and 3.5, respectively, *p* < 0.01; Fig. 5a).

The impact of mutations, chromosomal abnormalities, and clinical variables on outcome in CBF leukemia analyzed by univariate cox regression is shown in Supplementary Figs. S5 and S6. When analyzing the prognostic relevance of distinct mutations by multivariate cox regression, *KIT* D816, *DHX15,* and *SMC1A* mutations were independent prognostic factors (Fig. 5). Mutations in *DHX15* and *SMC1A* correlated with shorter OS in *RUNX1/RUNX1T1* leukemia (*DHX15*: HR = 3.57, *p* = 0.02; *SMC1A*: HR = 6.47, *p* < 0.001), while point mutations in *KIT* at position D816 correlated with reduced RFS in *RUNX1*/*RUNX1T1* leukemia (HR = 2.27, *p* = 0.046; Supplementary Fig. S7). In contrast to recent reports [8, 20, 21], the mutational burden of *KIT* D816 mutations was not relevant when assessing the prognostic impact of this mutation in our cohort (Supplementary Fig. S8). Considering the adverse prognostic relevance of *SMC1A* mutations, we evaluated alterations of genes encoding components of the cohesion complex together resulting in significantly shorter OS (HR = 1.93, *p* = 0.05) but not RFS (HR = 0.96, *p* = 0.8) in univariate analysis. These findings suggest that *SMC1A* has a unique prognostic relevance unlike the other cohesion genes.

Almost half of the CBF leukemia patients had additional chromosomal changes. Trisomy 22 was significantly associated with inv(16) leukemia, while a deletion of 9q and deletion of sex chromosomes correlated with t(8;21) leukemia. *CBFB*/*MYH11*-rearranged patients with trisomy 8 or trisomy 22 had a tendency toward longer RFS than patients without these trisomies (Fig. 5b). Loss of the Y-chromosome in male patients correlated with improved RFS in univariate analysis in *RUNX1/RUNX1T1* leukemia (HR = 0.39, *p* = 0.047; Supplementary Fig. S6). Due to the low patient number multivariate analysis could not be performed with regards to gender.

CR achievement rates were similarly high in the inv(16) cohort (97.1%) and the t(8;21) cohort (95.3%). Furthermore, no mutation or additional chromosomal aberration was associated with CR in either of the two cohorts.

### Mutation persistence or gain at CR

In 110 patients CR samples were available for detection of driver mutations, potentially indicating clonal hematopoiesis. We found persistence or gain of *DNMT3A* mutations in 4 out of 9 *DNMT3A*-mutated patients, with one patient harboring two different alterations of *DNMT3A* (Supplementary Table S4). Interestingly, we detected a gain of *TP53* mutations during CR in three CBF leukemia patients - all of these patients eventually relapsed. In one patient, the *TP53* mutation harboring clone expanded at relapse, while in the second patient the TP53 mutated clone was hardly detectable at relapse (Supplementary Table S4). For the third patient, appropriate sample material from the timepoint of relapse was not available.

## Discussion

We aimed to improve our understanding of CBF leukemia, a heterogeneous AML subtype, focusing on cooperating genetic events that might be responsible for the divergent clinical course. In the present study, we provide a comprehensive mutation catalog of 292 adult CBF leukemia patients and determined the prognostic relevance of mutations in distinct genes. A comparison of these intensively treated patients is conclusive even if they were treated in different trials, since all intensive AML chemotherapy protocols are similarly based on combinations of cytarabine and anthracyclines.

We found novel recurrent mutations, e.g., in *NFE2* and *MN1*. *NFE2* is a known hematopietic transcription factor. Recently, acquired *NFE2* mutations were described in 4 out of 6 patients with isolated myelosarcoma (MS) but were not found in 12 cases with MS and concomitant or previous AML [22]. Of note, one of these acquired mutations affected amino acid R272—the mutational hotspot detected in our CBF leukemia cohort. This region (AA 271–273) within the DBD encodes the nuclear localization sequence, critical for transcriptional activation of the NFE2 protein [23]. In 2–3% of myeloproliferative neoplasms cases, truncating *NFE2* mutations were identified and confer a proliferative advantage [24]. *MN1* is known as tumor suppressor gene [5]. Overexpression of *MN1* is an important cooperating event in inv(16) leukemia [25]. In AML with normal cytogenetics, *MN1* overexpression is associated with poor prognosis [26]. Of note, we identified hotspot mutations resulting in loss of the C-terminal part of *MN1*. Interestingly, it was shown that C-terminal deletion mutants of *MN1* are still able to cause leukemia in mice and confer higher engraftment rates [27]. Thus, the truncating mutations found in our patients (Fig. 4) likely result in gain-of-function.

In addition, we found recurrent mutations in *CSF3R* and *DHX15*. Acquisition of extracellular point mutations and mutations that truncate the cytoplasmic domain of *CSF3R* is a common phenomenon in severe congenital neutropenia (SCN) and chronic neutrophilic leukemia patients and leads to ligand-independent activation of the receptor and thus, to JAK-STAT, MAPK-ERK, and PI3K-AKT signaling pathway activation [28, 29]. SCN patients with *CSF3R* mutation are predisposed to AML and the mutation frequency increases up to 80% after leukemic transformation. *CSF3R* mutations have been reported in 2% of pediatric AML, where they were associated with *CEBPA* mutations and CBF-rearrangements [30]. So far, *CSF3R* aberrations were rarely found in adult de novo AML [29, 31]. A recent study from our research group confirmed the association of *CSF3R* alterations with biallelic *CEBPA* mutations also in adults [32]. *DHX15* mutations are common in *RUNX1*/*RUNX1T1* leukemia. *DHX15* is part of the splicing machinery and missense mutations at position R222 result in increased numbers of alternative splicing events [18, 33]. Of note, alterations in other spliceosome components are rare in CBF leukemia.

Our findings suggest that the mutatome of CBF leukemia is genetically complex with the co-existence of distinct subclones in more than half of the patients. In regards to recently published data on the prognostic impact of clonal interference in CBF leukemia [34], parallel evolution of more than one mutation in the signaling genes *KIT*, *RAS*, *FLT3*, *JAK1/2*, *CBL*, or *PTPN11* in a single patient (assuming that these mutations arise in independent subclones) did not affect overall or RFS in our CBF leukemia cohort (Supplementary Figs. S5 and S6).

While *CBFB*/*MYH11*-rearranged leukemias were associated with mutations in *RAS*-genes, *FLT3* and exon 8 frameshift mutations in *KIT* gene, *RUNX1/RUNX1T1*-rearranged leukemia correlated with mutations in *KIT* D816, epigenetic modifiers (*ASXL1/2, TET2, IDH2, DNMT3A*), cohesion complex components (*RAD21, SMC3, SMC1A, STAG2*), *ZBTB7A*, *GATA2*, *JAK2/3* and *DHX15*. These recurrent mutations likely represent other pathways that are relevant for *RUNX1/RUNX1T1*-initiated leukemogenesis. Of note, most of these mutations have been associated with unfavorable prognosis in AML in recent studies [14, 16, 17, 35–38]. In our *RUNX1/RUNX1T1* cohort, aberrations in *DHX15 and SMC1A* were found to be strong and independent adverse prognostic factors for OS and RFS.

*SMC1A* mutations were found mostly with high allelic frequency, likely representing the predominant clone. Although *SMC1A* is localized on the X chromosome, its alterations are neither significantly associated with X loss nor with gender in our cohort (Supplementary Fig. S9).

In the present study, *TET2* alterations were mutually exclusive with mutations in genes of the cohesion complex and *ZBTB7A*. Instead they often co-occur with signaling mutations. *TET2* alterations were commonly found in the predominant leukemic clone suggesting an early lesion and a potential initiating event during leukemogenesis. The tendency toward unfavorable prognosis in patients with *TET2* mutation is based on the strong correlation with older age (HR = 4.25; Fig. 3). *TET2* mutations were not found in patient younger than 40 years (Supplementary Fig. S10).

Compared with recently published whole-exome, whole-genome, and targeted sequencing data of heterogeneous cohorts of adult and pediatric CBF AML patients from two different research groups [8, 18], we observed a higher prevalence of alterations in signaling pathway genes, in *ZBTB7A*, *JAK* and epigenetic modifiers, a discrepancy partially explained by the often low VAF of signaling pathway mutations and the deeper sequencing coverage achieved by our targeted sequencing approach. A second potential bias might be that in both studies pooled data of adult and childhood CBF AML were analyzed. As observed by the authors themselves, adult and pediatric patients with CBF AML differ in the pattern of mutations which are dependent on age (*TET2*, *KIT*, *EZH2*, *KDM6A,* and *FLT3*) [35, 39]. A further limitation was mentioned by Faber et al. themselves: despite adequate sequence coverage in their WES cohort they did not identify mutations in *ASXL1* [18]. Moreover, the study by Duployez et al. was limited to mutational hotspots in 40 genes, recurrently mutated in myeloid malignancies [8]. Thus, they missed novel mutations in genes like *ZBTB7A*, *MN1,* and *NFE2*, as well as novel mutational hotspots like N676K in exon 16 of *FLT3*.

A limitation of our study is that we focused on a defined set of known and novel genes. The entire coding sequence (exome) was analyzed only for 12 CBF patients. This might be the reason why we missed novel recurring but rare mutations in *RUNX1/RUNX1T1*-rearranged leukemia [*CCND2* (6–12%) [18, 40], *MGA* (8%) [18]]. However, whole-exome or whole-genome sequencing requires matched germline samples, and suitable DNA specimens were not available for all of our patients.

In summary, our study provides a detailed overview of cooperating mutations in one of the largest adult CBF leukemia cohorts so far and highlights fundamental differences between *CBFB/MYH11*- and *RUNX1/RUNX1T1*-rearranged leukemia. Thus, they seem to represent distinct clinicobiological entities. We provide detailed insights into the prognostic relevance of several cooperating mutations and found a significant impact on survival for mutations in *KIT*, *DHX15*, and *SMC1A* and loss of the Y-chromosome. Our findings have implications for the use of already established targeted therapies aimed at eradicating malignant clones carrying mutations in *FLT3*, *KIT*, *JAK2*, *TET2*, *DNMT3A,* and *IDH1/2*. Furthermore, the unfavorable genetic profiles in CBF leukemia patients could be considered for future risk stratification. Especially for the patients with high risk of relapse, the clinical mutatome of CBF leukemia has a strong potential for translation into alternative treatment strategies.

## Supplementary information

Supplemental Information
