# Supplementary material for: The clinical mutatome of core binding factor leukemia
Source: Leukemia. 2020 Jan 2;34(6):1553–62. doi: 10.1038/s41375-019-0697-0 (PMC7266744; doi:10.1038/s41375-019-0697-0)

SUPPLEMENTAL INFORMATION

THE CLINICAL MUTATOME OF CORE BINDING FACTOR LEUKEMIA

Sabrina Opatz[1,2,3,4], Stefanos A. Bamopoulos[1], Klaus H Metzeler[1,2,3,4], Tobias Herold[1], Bianka Ksienzyk[1], Kathrin Bräundl[1,2,3,4], Sebastian Tschuri[1,3,4], Sebastian Vosberg[2], Nikola P Konstandin[1], Christine Wang[1], Luise Hartmann[1,2,3,4], Alexander Graf[5], Stefan Krebs[5], Helmut Blum[5], Stephanie Schneider[1,6], Christian Thiede[3,4,7], Jan Moritz Middeke[3,4,7], Friedrich Stölzel[3,4,7], Christoph Röllig[3,4,7], Johannes Schetelig[3,4,7], Gerhard Ehninger[3,4,7], Alwin Krämer[3,4], Jan Braess[8], Dennis Görlich[9], Maria Cristina Sauerland[9], Wolfgang E. Berdel[10], Bernhard J. Wörmann[11], Wolfgang Hiddemann[1,2,3,4], Karsten Spiekermann[1,2,3,4], Stefan K Bohlander[12], Philipp A Greif[1,2,3,4]

1 Laboratory for Leukemia Diagnostics, Department of Medicine III, University Hospital, LMU Munich, Munich, Germany
2 Experimental Leukemia & Lymphoma Research, Department of Medicine III, University Hospital, LMU Munich, Munich, Germany
3 German Cancer Consortium (DKTK), and
4 German Cancer Research Center (DKFZ), Heidelberg, Germany
5 Laboratory for Functional Genome Analysis at the Gene Center, LMU Munich, Munich, Germany;
6 Institute of Human Genetics, University Hospital, LMU Munich, Munich, Germany
7 Department of Internal Medicine 1, University Hospital Carl Gustav Carus, Dresden, Germany
8 Oncology and Hematology, St. John of God Hospital, Regensburg, Germany
9 Institute of Biostatistics and Clinical Research, University of Münster, Münster, Germany
10 Department of Medicine A, Hematology, Oncology and Pneumology, University of Münster, Münster, Germany
11 Department of Hematology, Oncology and Tumor Immunology, Charité University Medicine, Campus Virchow, Berlin, Germany
12 Department of Molecular Medicine and Pathology, Faculty of Medical and Health Sciences, University of Auckland, New Zealand

Correspondence:
Philipp A. Greif, MD
Max Lebsche Platz 30
81377 München
Phone: +49 89 4400 43982
FAX: +49 89 4400 43970
pgreif@med.uni-muenchen.de

Supplementary Table S1: Table of targeted genes. Design of the custom targeted gene panel (HaloPlex®; Agilent Technologies, Santa Clara, CA), comprising 129 candidate genes.

TargetID	Interval	Regions	Size (bp)	Coverage (%)	HighCoverage	LowCoverage
ADIPOR2	chr12:1863500-1895248	7	1301	99.23	7	0
ANKRD26	chr10:27283989-27389265	37	6404	99.84	37	0
ASXL1	chr20:30946569-31025151	17	5060	100.00	17	0
ASXL2	chr2:25964888-26101101	13	4571	100.00	13	0
BCOR	chrX:39909159-39937192	15	5648	100.00	15	0
BCORL1	chrX:129139198-129190121	14	5690	100.00	14	0
BRAF	chr7:140426284-140624513	21	2799	100.00	21	0
CACNA1C	chr12:2080219-2800375	54	8412	100.00	54	0
CALR	chr19:13049484-13054805	10	1494	100.00	10	0
CAT	chr11:34460551-34492990	13	1844	100.00	13	0
CBL	chr11:119077118-119170501	16	3041	99.90	16	0
CEBPA	chr19:33792234-33793435	1	1202	100.00	1	0
CELF3	chr1:151677507-151689284	14	1726	100.00	14	0
CLEC2A	chr12:10051493-10084938	6	718	100.00	6	0
CSF3R	chr1:36931687-36945107	16	3018	100.00	16	0
CSMD1	chr8:2796097-4851948	73	12252	99.62	72	1
DDR1	chr6:30852987-30867869	19	3383	99.79	19	0
DDR2	chr1:162688844-162750046	16	3064	100.00	16	0
DHX15	chr4:24529537-24586026	14	2668	100.00	14	0
DNMT3A	chr2:25457138-25536863	25	3388	100.00	25	0
DNMT3B	chr20:31367912-31395719	23	3058	100.00	23	0
EP300	chr22:41488999-41574970	31	7865	99.91	31	0
EPB41L4A	chr5:111500677-111754746	23	2521	100.00	23	0
EPHA8	chr1:22890119-22928244	17	3531	100.00	17	0
ETV6	chr12:11803052-12044545	10	1643	100.00	10	0
EZH2	chr7:148504728-148544400	21	2876	96.56	20	1
FAT1	chr4:187509736-187630991	29	14593	99.88	29	0
FAT3	chr11:92085269-92624289	28	14488	100.00	28	0
FAT4	chr4:126237557-126412933	18	15440	99.99	18	0
FBXW7	chr4:153244023-153332965	14	2898	100.00	14	0
FCN1	chr9:137801634-137809727	9	1161	100.00	9	0
FLT3	chr13:28578179-28674657	25	3504	100.00	25	0
GATA1	chrX:48649507-48652685	5	1446	100.00	5	0
GATA2	chr3:128199852-128205884	5	1543	99.55	5	0
GATA3	chr10:8097609-8115996	5	1435	100.00	5	0
GK	chrX:30671645-30746869	25	2490	100.00	25	0
GLDC	chr9:6533007-6645509	25	3563	100.00	25	0
GPX8	chr5:54456011-54460056	3	690	100.00	3	0
GSPT1	chr16:11966966-12009587	16	2265	100.00	16	0
GTPBP8	chr3:112709837-112719776	7	998	100.00	7	0
HECW2	chr2:197065991-197298157	28	5279	100.00	28	0
HERC1	chr15:63901270-64067832	79	16255	99.91	79	0
HRAS	chr11:532626-534332	5	733	100.00	5	0
IDH1	chr2:209101793-209116285	8	1405	100.00	8	0
IDH2	chr15:90627488-90645632	11	1579	100.00	11	0
INHBC	chr12:57828660-57843815	2	1099	100.00	2	0
ITGAL	chr16:30484149-30532996	31	4386	100.00	31	0
JAK1	chr1:65300235-65351957	24	3945	100.00	24	0
JAK2	chr9:5021978-5126801	23	3859	100.00	23	0
JAK3	chr19:17937542-17955236	23	3913	100.00	23	0

TargetID	Interval	Regions	Size (bp)	Coverage (%)	HighCoverage	LowCoverage
KAZN	chr1:14925484-15441141	16	2900	100.00	16	0
KCNA4	chr11:30032254-30034235	1	1982	100.00	1	0
KCNAB1	chr3:155836090-156254546	18	2216	100.00	18	0
KCNH4	chr17:40312048-40332973	16	3374	100.00	16	0
KCNJ4	chr22:38822790-38824147	1	1358	100.00	1	0
KDM2A	chr11:66888778-67022536	21	4071	99.85	21	0
KDM6A	chrX:44732788-44970666	31	5090	100.00	31	0
KIT	chr4:55524172-55604733	21	3351	99.97	21	0
KITLG	chr12:88898963-88974065	9	1002	100.00	9	0
KLHL14	chr18:30254610-30352057	10	2266	100.00	10	0
KMT2A	chr11:118307218-118392897	38	12842	99.33	38	0
KMT2C	chr7:151833907-152132881	60	16122	99.16	57	3
KMT2D	chr12:49415553-49449117	55	17762	98.78	54	1
KRAS	chr12:25362719-25398328	6	828	96.74	5	1
MECOM	chr3:168802687-169381170	20	4216	100.00	20	0
MEIS1	chr2:66662979-66798671	21	2734	100.00	21	0
MEIS2	chr15:37184364-37391677	15	1938	100.00	15	0
MN1	chr22:28146893-28196541	2	4003	98.95	2	0
MTFR1L	chr1:26149586-26158527	8	1219	100.00	8	0
MYD88	chr3:38180143-38182787	5	1054	100.00	5	0
MYH1	chr17:10395723-10419969	38	6580	100.00	38	0
MYO5B	chr18:47352831-47721173	40	6347	100.00	40	0
NFAT5	chr16:69600195-69729284	16	5077	100.00	16	0
NFE2	chr12:54686148-54689042	2	1162	100.00	2	0
NID2	chr14:52472194-52535722	22	4568	100.00	22	0
NOTCH1	chr9:139390513-139440248	34	8348	100.00	34	0
NPM1	chr5:170814943-170837579	12	1134	100.00	12	0
NRAS	chr1:115251146-115258791	4	650	100.00	4	0
OR4X1	chr11:48285403-48286340	1	938	100.00	1	0
OR5D18	chr11:55587096-55588057	1	962	100.00	1	0
PCDHAC2	chr5:140346342-140389532	4	3194	100.00	4	0
PDE4B	chr1:66378988-66838371	20	3073	100.00	17	0
PHF6	chrX:133511638-133559370	9	1387	100.00	9	0
PTCRA	chr6:42883798-42893430	5	1034	100.00	5	0
PTEN	chr10:89623697-89725239	9	1912	100.00	9	0
PTPN11	chr12:112856906-112942578	16	2142	100.00	16	0
RAD21	chr8:117859729-117878978	13	2156	100.00	13	0
RNF216	chr7:5662481-5800710	18	3323	98.86	17	1
RPL7	chr8:74203269-74205857	6	867	100.00	6	0
RUNX1	chr21:36164422-36421206	11	1804	99.94	11	0
RYR2	chr1:237205812-237995957	110	17206	99.60	108	2
SCAND3	chr6:28539678-28554504	4	4058	100.00	4	0
SF1	chr11:64532851-64545874	14	2733	100.00	14	0
SF3A1	chr22:30730573-30752791	16	2702	100.00	16	0
SF3B1	chr2:198257017-198299733	27	4585	98.93	26	1
SLC6A3	chr5:1394840-1443322	15	2315	100.00	15	0
SMC1A	chrX:53407014-53449559	26	4322	100.00	26	0
SMC3	chr10:112327565-112364070	29	4234	99.41	28	1
SNRPA	chr19:41257304-41271082	7	1007	100.00	7	0
SPAG16	chr2:214149198-215275049	21	2907	99.66	21	0
SRP72	chr4:57333792-57368037	19	2449	99.43	19	0
SRSF2	chr17:74732233-74733252	2	706	100.00	2	0
STAG1	chr3:136057086-136349750	36	4654	99.66	35	1
STAG2	chrX:123156468-123234457	34	4541	100.00	34	0
STAT5A	chr17:40441420-40462697	19	2771	100.00	19	0
STAT5B	chr17:40353746-40384155	18	2724	100.00	18	0
TERT	chr5:1253833-1295114	16	3719	100.00	16	0
TET1	chr10:70332086-70451581	11	6631	100.00	11	0
TET2	chr4:106111617-106197686	10	6365	100.00	10	0
TET3	chr2:74213521-74329313	12	5652	100.00	12	0
TEX13A	chrX:104463636-104465091	2	1272	97.80	2	0
TGM6	chr20:2361605-2413299	13	2381	100.00	13	0
TLE1	chr9:84199103-84303165	21	2817	100.00	21	0
TLE2	chr19:2997647-3045796	22	2842	100.00	22	0
TLE3	chr15:70342426-70389368	23	3337	100.00	23	0
TLE4	chr9:82187696-82340069	27	3352	99.73	27	0
TLN2	chr15:62939500-63132819	56	8872	100.00	56	0
TMEM144	chr4:159133810-159174689	11	1386	100.00	11	0
TMEM59L	chr19:18723759-18731356	8	1189	100.00	8	0
TP53	chr17:7565247-7579922	14	1658	94.63	13	1
U2AF1	chr21:44513202-44527614	9	970	100.00	9	0
U2AF2	chr19:56166461-56185444	14	1821	100.00	14	0
USP9X	chrX:40982872-41091787	45	8714	100.00	45	0
WBP1L	chr10:104503801-104573098	5	1219	100.00	5	0
WDR70	chr5:37379460-37752685	19	2417	100.00	19	0
WT1	chr11:32410594-32456901	11	1788	100.00	11	0
ZBTB7A	chr19:4047740-4055240	2	1795	100.00	2	0
ZFHX4	chr8:77616314-77776811	13	11216	99.54	13	0
ZRSR2	chrX:15808609-15841375	12	1930	93.01	11	1

Supplementary Table S2: Metrics summary of the exome data.

Diagnostic AML samples:

UPN	1	2	3	4	5	6	7	8	9	10	11	12
# of total reads:	52803026	50607320	75540920	58662750	81508126	40812262	74996526	57345902	44252806	74706896	75764126	68389502
% of reads mapped to genome:	98.6167	98.3985	99.3901	98.2539	98.682	98.8155	99.076	98.5911	93.3704	98.6926	99.1994	99.0666
% of reads mapped with MQ >= Q20:	88.64	87.6	86.17	86.63	87.53	89.37	88.95	88.14	92.22	89.09	97.13	97.08
# of sequenced bases mapped to genome:	4320052285	4131856844	5375597491	5357487090	6345518697	3194451035	6149962302	4715869171	3253808788	6753353028	7498763121	6769866831
% of bases mapped in HQ reads:	89.92	89.07	86.74	88.24	88.78	90.5	89.84	89.45	98.79	90.36	97.92	98
% of >=Q20 bases mapped in HQ reads:	88.84	88.19	85.89	87.34	86.79	87.43	88.04	88.56	97.83	89.11	95.06	95.77
% of target sequenced:	95.9	95.14	96.03	94.69	96.59	92.36	95.84	95.35	95.51	95.97	97.94	98.01
% of target sequenced, min 10x coverage:	85.16	82.94	87.62	82.12	87.9	70.39	85.09	84.44	77.63	82.95	95.85	95.39
mean target coverage:	46.61	49.78	64.68	64.47	72.27	32.14	67.46	53.36	33	57.49	96.54	86.05
% bases on target:	41.02	45.95	47.26	46.09	43.99	36.79	41.75	43.01	35.22	32.26	46.01	45.44
% bases near target (+/- 250 nt):	33.63	28.03	31.56	26.48	25.07	26.36	29.04	29.73	36.02	31.92	29.4	29.05
% bases on or near target:	74.66	73.97	78.83	72.57	69.06	63.15	70.8	72.74	71.24	64.18	75.42	74.49

Remission samples:

UPN	1	2	3	4	5	6	7	8	9	10	11	12
# of total reads:	62175578	55014740	73126312	67110342	82693346	82584192	79314698	39106644	37051134	40218274	73098222	76653330
% of reads mapped to genome:	98.7213	98.633	99.373	98.2388	98.7426	98.9636	99.099	98.2097	99.4887	99.5735	99.0988	99.1015
% of reads mapped with MQ >= Q20:	88.2	88.1	86.14	88.54	87.39	88.61	88.91	88.82	98.18	98.39	97.07	97.01
# of sequenced bases mapped to genome:	5068381059	4516729486	5212685664	6043586892	6457691850	6514886214	6458764515	3210303795	2904047926	3150798239	7225689271	7591339875
% of bases mapped in HQ reads:	89.4	89.37	86.73	90.22	88.58	89.59	89.8	90.49	98.7	98.83	97.96	97.9
% of >=Q20 bases mapped in HQ reads:	88.12	88.52	85.95	89.06	86.63	87.88	87.99	89.75	97.77	97.82	94.97	95.65
% of target sequenced:	95.17	95.47	96.23	95.99	96.51	96.24	95.48	95.45	95.44	95.66	97.95	98.06
% of target sequenced, min 10x coverage:	84.14	84.29	87.77	82.99	88.26	86.69	84.15	81.55	75.05	78.65	95.76	95.93
mean target coverage:	59.1	52.4	61.47	51.01	74.76	69.75	70.92	34.24	28.92	32.79	92.16	96.93
% bases on target:	44.31	44.24	46.38	31.97	44.79	40.95	41.69	40.17	34.6	36.21	45.57	45.71
% bases near target (+/- 250 nt):	28.27	28.53	31.33	31.19	26.38	30.39	30.9	30.29	35.48	37.17	30.41	29.75
% bases on or near target:	72.59	72.76	77.71	63.16	71.16	71.34	72.59	70.46	70.07	73.38	75.99	75.46

Supplementary Table S3: Summary of the results from exome sequencing of 12 patients with CBF leukemia. Asterisk indicates subclonal mutations, which were missed by exome sequencing and detected by targeted amplicon sequencing. Estimated VAF-Range ± 2 standard deviation (SD) assigned according to targeted amplicon sequencing.

UPN 72	Gene_name	AA_change	Position (hg19)	Reference	Change	Effect	Var Freq	VAF range ±2SD
	PDE4B	L395*	chr1:66829154	T	A	stop_gained	37,9%	19,9-56,0
	WDR70	D380N	chr5:37697802	G	A	Missense	36,7%	25,9-47,6
	RPL7	A204S	chr8:74203416	C	A	Missense	36,1%	25,6-46,7
	GPX8	K68N	chr5:54456224	A	C	Missense	34,8%	14,9-54,6
	INHBC	R323H	chr12:57843714	G	A	Missense	33,3%	11,1-55,6
	NRAS	G12D	chr1:115258747	C	T	Missense	25,5%	13,3-37,7
**UPN 48**	**Gene_name**	**AA_change**	**Position (hg19)**	**Reference**	**Change**	**Effect**	**Var Freq**	**VAF range ±2SD**
	CAT	A251V	chr11:34477598	C	T	Missense	52,5%	39,5-65,5
	FLT3	N676K	chr13:28602340	G	T	Missense	20,6%	16,6-24,6
	*FLT3	p.836_837del	chr13:28592635-7	*	minus CAT	Deletion	5,8%	4,0-7,7
	*FLT3	S451F	chr13:28610138	G	A	Missense	5,2%	2,3-8,2
**UPN 54**	**Gene_name**	**AA_change**	**Position (hg19)**	**Reference**	**Change**	**Effect**	**Var Freq**	**VAF range ±2SD**
	TMEM144	N3I	chr4:159133827	A	T	Missense	34,0%	24,3-43,8
	MYH1	A1682D	chr17:10399391	G	T	Missense	55,6%	51,7-59,6
	MYO5B	R63W	chr18:47566636	G	A	Missense	48,5%	44,0-53,0
	FCN1	N117S	chr9:137804980	T	C	Missense	48,5%	43,7-53,3
	ZFHX4	R2915H	chr8:77767949	G	A	Missense	39,7%	36,4-43,0
	KCNAB1	C28Y	chr3:155861050	G	A	Missense	43,3%	39,1-47,5
	KIT	Y418X	chr4:55589772	C	A	Missense	43,6%	39,0-47,7
	KIT	D419A	chr4:55589774	A	C	Missense	43,3%	39,0-47,7
	KIT	p.417_418del	chr4:55589769-71	*	minus TT	frameshift	43,0%	39,0-47,7
	*NRAS	G12A	chr1:115258747	C	G	Missense	9,3%	6,7-11,9
**UPN 86**	**Gene_name**	**AA_change**	**Position (hg19)**	**Reference**	**Change**	**Effect**	**Var Freq**	**VAF range ±2SD**
	GK	R413Q	chrX:30738757	G	A	Missense	76,3%	69,9-82,7
	KCNJ4	T297M	chr22:38823248	G	A	Missense	48,6%	31,7-65,5
	CELF3	G31A	chr1:151688405	C	G	Missense	50,9%	41,5-60,3
	KRAS	G12D	chr12:25398284	C	T	Missense	35,8%	25,2-46,5
	NFE2	S188C	chr12:54686717	G	C	Missense	39,0%	34,1-44,0
	KCNH4	G558R	chr17:40318483	C	T	Missense	41,0%	36,2-45,7
**UPN 60**	**Gene_name**	**AA_change**	**Position (hg19)**	**Reference**	**Change**	**Effect**	**Var Freq**	**VAF range ±SD**
	MTFR1L	R108H	chr1:26153189	G	A	Missense	46,8%	43,1-50,1
	NID2	G588S	chr14:52508886	C	T	Missense	39,7%	34,0-45,3
	WBP1L	G193W	chr10:104572636	G	T	Missense	34,2%	29,1-39,3
	HERC1	Q1784*	chr15:63986641	G	A	stop_gained	36,4%	31,5-41,3
	OR5D18	V135G	chr11:55587509	T	G	Missense	17,8%	12,8-22,9
	KAZN	R147G	chr1:15361265	C	G	Missense	18,8%	13,6-23,9
	*KRAS	G12D	chr12:25398284	C	T	Missense	11,0%	3,6-18
**UPN 61**	**Gene_name**	**AA_change**	**Position (hg19)**	**Reference**	**Change**	**Effect**	**Var Freq**	**VAF range ±2SD**
	WT1	R353fs	chr11:32417942	*	plus G	frameshift	45,4%	41,8-49,1
	KCNA4	R225C	chr11:30033553	G	A	Missense	34,2%	29,9-38,5
	TET1	T967M	chr10:70405386	C	T	Missense	31,4%	25,5-37,2
	GSPT1	R272X	chr16:11981570	G	A	stop_gained	49,4%	44,5-54,3
	MECOM	V363L	chr3:168834009	C	G	Missense	43,4%	35,7-51,1
	OR4X1	V269F	chr11:48286217	G	T	Missense	35,7%	28,9-42,6
	NRAS	Q61K	chr1:115256530	G	T	Missense	31,1%	28,8-33,5
	SPAG16	Q302K	chr2:214239805	C	A	Missense	43,7%	39,8- 47,6
	RNF216	R464RHCR	chr7:5760745	*	plus CTACAATGT	Insertion	48,7%	44,7-52,8
	*KRAS	G12V	chr12:25398284	C	A	Missense	4,0%	1,5-6,9
**UPN 126**	**Gene_name**	**AA_change**	**Position (hg19)**	**Reference**	**Change**	**Effect**	**Var Freq**	**VAF range ±2SD**
	CACNA1C	T79M	chr12:2224576	C	T	Missense	38,4%	35,8-40,9
	NRAS	Q61K	chr1:115256530	G	T	Missense	35,2%	32,0-38,2
	NFAT5	I1023V	chr16:69727077	A	G	Missense	43,6%	40,2-46,9
	TMEM59L	A142T	chr19:18726800	G	A	Missense	41,2%	37,3-45,2
	ADIPOR2	R169C	chr12:1889658	C	T	Missense	39,3%	33,6-45,1
	SLC6A3	R60P	chr5:1443134	C	G	Missense	39,2%	36,2-42,2
**UPN 129**	**Gene_name**	**AA_change**	**Position (hg19)**	**Reference**	**Change**	**Effect**	**Var Freq**	**VAF range ±2SD**
	SNRPA	R243Q	chr19:41270951	G	A	Missense	36,9%	34,1-39,7
	EPB41L4A	I674T	chr5:111500727	A	G	Missense	36,3%	28,9-43,6
	GTPBP8	N91K	chr3:112710119	C	A	Missense	36,8%	33,6-40,1
	GLDC	G287E	chr9:6605132	C	T	Missense	35,1%	32,3-37,8
	*FLT3	D835	chr13:28592641	T	A	Missense	3,0%	2,8-3,9
**UPN 81**	**Gene_name**	**AA_change**	**Position (hg19)**	**Reference**	**Change**	**Effect**	**Var Freq**	**VAF range ±2SD**
	HECW2	P632H	chr2:197183719	G	T	Missense	38,3%	31,9-44,8
	TGM6	E131K	chr202376049	G	A	Missense	40,7%	35,2-46,1
	KRAS	G12V	chr12:25398284	C	A	Missense	42,6%	34,2-50,9
**UPN 59**	**Gene_name**	**AA_change**	**Position (hg19)**	**Reference**	**Change**	**Effect**	**Var Freq**	**VAF range ±2SD**
	CLEC2A	I38M	chr12:10078884	A	C	Missense	40,0%	26,1-53,9
	KIT	Y418fs	chr4:55589772	*	plus TG	Insertion	39,8%	35,1-44,5
	KIT	D419E	chr4:55589775	C	A	Missense	40,3%	35,6-45,0
	KIT	p.420_421del	chr4:55589779	GC	*	Deletion	40,0%	35,1-44,5
	NRAS*	G13D	chr1:115258744	G	D	Missense	5,26%	2,0-8,5
**UPN 158**	**Gene_name**	**AA_change**	**Position (hg19)**	**Reference**	**Change**	**Effect**	**Var Freq**	**VAF range ±2SD**
	PTCRA	L57F	chr6:42890875	C	T	Missense	40,7%	34,9-45,9
	KIT	D812V	chr4:55599321	A	T	Missense	28,3%	23,8-34,2
	CSMD1	N3542K	chr8:2796176	G	C	Missense	48,2%	33,1-41,9
	PCDHAC2	R751M	chr5:140348603	G	T	Missense	37,2%	20,9-46,9
	KLHL14	R336W	chr18:30321954	G	A	Missense	45,6%	25,4-37,9
	SCAND3	S1031A	chr6:28540575	A	C	Missense	46,8%	22,0-41,1
	EPHA8	R198C	chr1:22903142	C	T	Missense	34,0%	25,6-34,1
	TEX13A	T281M	chrX:104464034	G	A	Missense	44,9%	38,3-47,8
	ITGAL	R281C	chr16:30495266	C	T	Missense	29,7%	27,6-37,0
	RYR2	R1610X	chr1:237774206	C	T	stop_gained	34,5%	35,0-44,6
	ZBTB7A	A175fs	chr19:4054708	C	plus C	frameshift	45,7%	40,9-49,6
	USP9X	N1895fs	chrX:41075505	T	plus CA	frameshift	44,6%	25,6-39,3
	NRAS*	G13D	chr1:115258744	C	T	Missense	6,0%	2,9-9,1
**UPN 8**	**Gene_name**	**AA_change**	**Position (hg19)**	**Reference**	**Change**	**Effect**	**Var Freq**	**VAF range ±2SD**
	ZBTB7A	R402H	chr19:4054026	C	T	Missense	81,2%	78,0-84,5

Supplementary Table S4: Mutation persistence or gain at CR.

UPN	Gene	Variant	VAF at diagnosis	variant reads	total reads	VAF at CR	variant reads	total reads	VAF at relapse	variant reads	total reads	VAF at 2nd CR	variant reads	total reads
173	**DNMT3A**	Y528X	**0,43**	112	259	**0,07**	25	343	**NA**	NA	NA	**NA**	NA	NA
110	**DNMT3A**	Q384fs	**0,50**	150	302	**NA**	NA	NA	**0,10**	15	139	**0,05**	7	347
110	**DNMT3A**	R409X	**0,01**	2	336	**NA**	NA	NA	**0,11**	26	231	**0,11**	46	427
259	**DNMT3A**	R882H	**0,00**	0	1134	**0,01**	5	963	**0,07**	55	787	**NA**	NA	NA
117	**DNMT3A**	G361R	**0,04**	10	225	**0,31**	66	214	**NA**	NA	NA	**NA**	NA	NA
265	**TP53**	H154R	**0,00**	0	956	**0,12**	77	619	**0,93**	717	774	**NA**	NA	NA
271	**TP53**	R136H	**0,00**	0	1088	**0,09**	89	1010	**0,01**	7	908	**NA**	NA	NA
103	**TP53**	C203S	**0,00**	4	1431	**0,11**	60	547	**NA**	NA	NA	**NA**	NA	NA

Supplementary Figure S1: Impact of HSCT in first CR on outcome in CBF leukemia. Kaplan-Meier estimates indicate overall survival and relapse free survival in CBF leukemia for allogeneic HSCT in first CR. Number of patients in each group is shown in parentheses. P values were determined by log rank test.

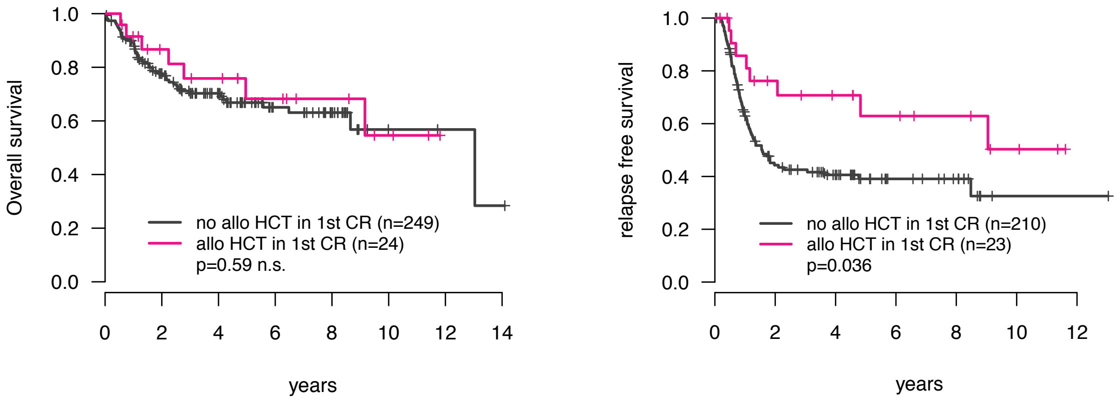

Supplementary Figure S2: Somatic mutation profile of 12 CBF leukemia patients. Exome sequencing results of 12 patients with CBF leukemia. Mutations were clustered (circled) when detected with similar VAF-range (+/- 2 SD). 8 out of 12 patients were oligoclonal. In patient #48, #60 and #129 known AML driver genes are present only in a subclone.

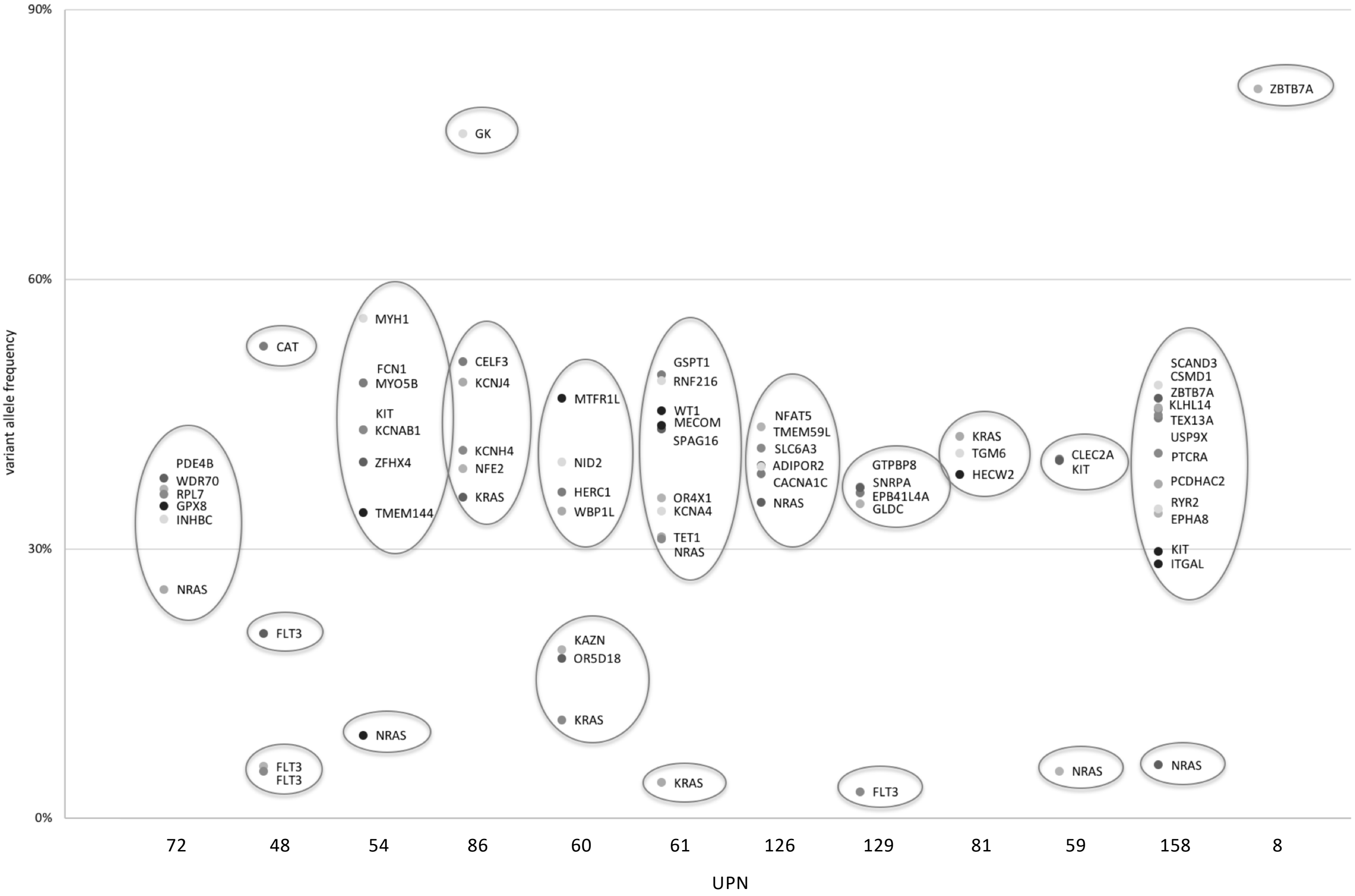

Supplementary Figure S3: Results from targeted amplicon sequencing in 25 patients with CBFB/MYH11-rearrangement (left), and 8 patients with RUNX1/RUNX1T1-rearrangement (right) where peripheral blood was used. Each column represents one patient, each line shows the status of the indicated genetic aberrations. The color code is described in the figure legend. Range of the PB blast count was 6-93% (Median: 40%, Mean: 45.6%). Also in samples with low blast count, common mutations could be identified.

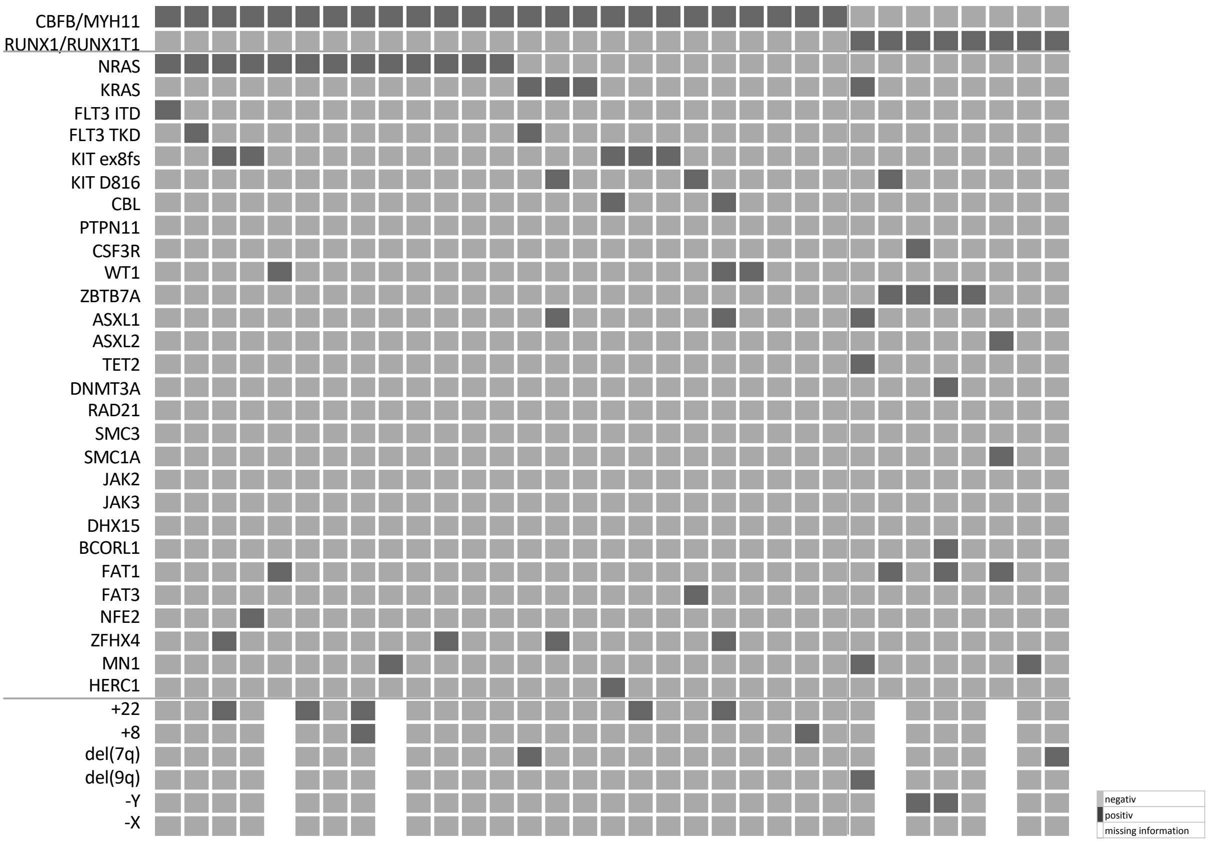

Supplementary Figure S4: Survival in CBFB/MYH11- vs. RUNX1/RUNX1T1-Leukemia. Kaplan-Meier estimates indicate overall survival and relapse free survival. Number of patients in each group is shown in parentheses. P values were determined by log rank test.

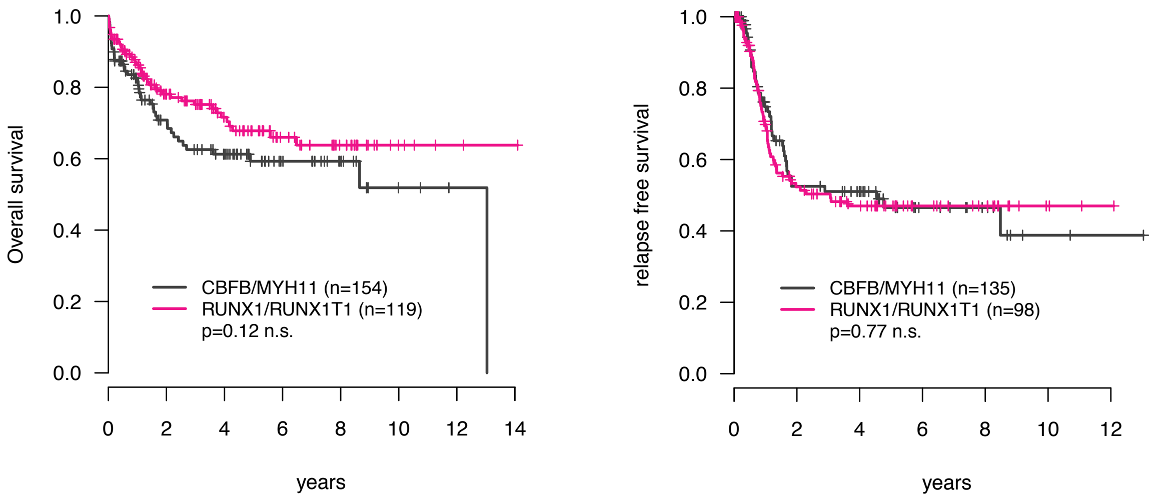

Supplementary Figure S5: Impact of different mutations and clinical variables on outcome in *CBFB/MYH11*-rearranged leukemia. Univariate Cox Regression. **A** Overall survival in patients with inv(16). **B** Relapse-free survival in patients with inv(16). Hazard ratios (HR), with corresponding Wald-test p-values and 95% confidence intervals (CI) are reported. P values are considered significant at p≤ 0.1.

A

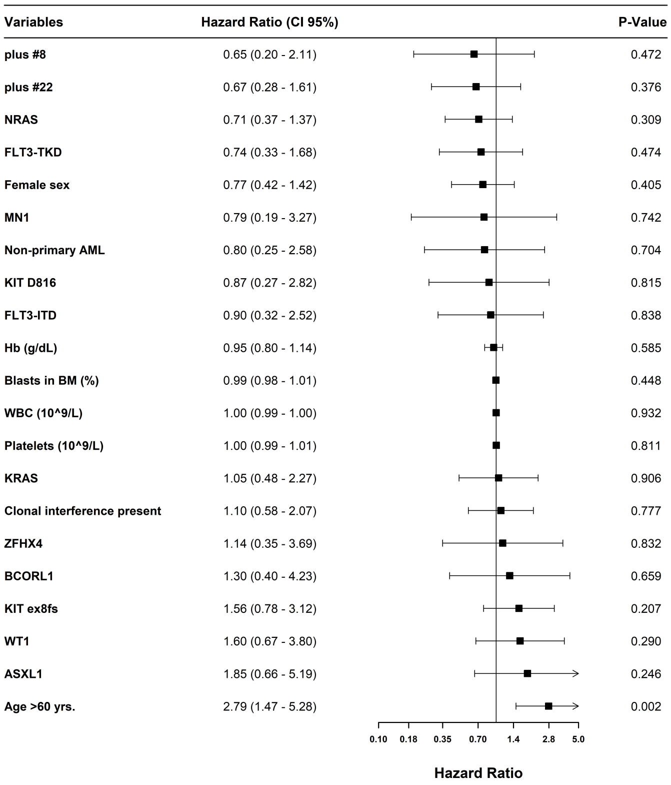

B

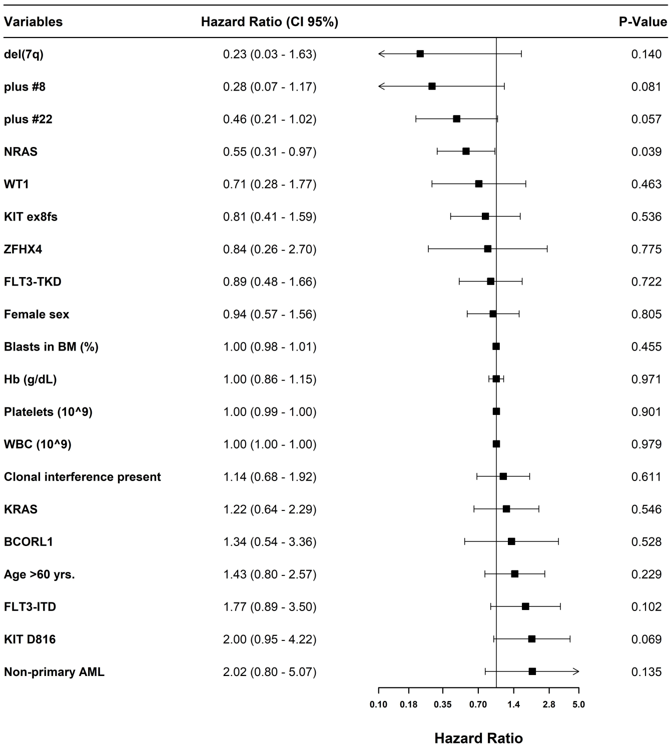

Supplementary Figure S6: Impact of different mutations and clinical variables on outcome in *RUNX1/RUNX1T1*-rearranged leukemia. Univariate Cox Regression. **A** Overall survival in patients with t(8;21). **B** Relapse-free survival in patients with t(8;21). Hazard ratios (HR), with corresponding Wald-test p-values and 95% confidence intervals (CI) are reported. P values are considered significant at p≤ 0.1.

A

Variables	Hazard Ratio (CI 95%)	P-Value
ASXL1	0.48 (0.15 - 1.56)	0.221
del(Y) (males only)	0.50 (0.19 - 1.29)	0.153
del(Y)	0.62 (0.28 - 1.38)	0.242
del(9q)	0.68 (0.24 - 1.94)	0.472
FAT3	0.72 (0.17 - 2.99)	0.652
Female sex	0.80 (0.43 - 1.48)	0.482
FLT3-ITD	0.82 (0.25 - 2.67)	0.745
ZBTB7A	0.84 (0.40 - 1.77)	0.653
JAK3	0.85 (0.30 - 2.38)	0.752
FAT1	0.86 (0.27 - 2.81)	0.809
Clonal interference present	0.87 (0.38 - 1.95)	0.727
Hb (g/dL)	0.92 (0.80 - 1.06)	0.247
NRAS	0.95 (0.40 - 2.27)	0.915
ASXL2	0.96 (0.49 - 1.89)	0.906
FLT3-TKD	0.96 (0.30 - 3.12)	0.948
Platelets (10^9/L)	0.99 (0.97 - 1.00)	0.036
WBC (10^9/L)	1.00 (0.99 - 1.02)	0.681
Blasts in BM (%)	1.01 (0.99 - 1.02)	0.362
plus #8	1.06 (0.25 - 4.45)	0.936
KRAS	1.06 (0.26 - 4.42)	0.934
JAK2	1.29 (0.51 - 3.29)	0.595
del(X)	1.36 (0.61 - 3.02)	0.450
del(X) (females only)	1.43 (0.51 - 4.00)	0.495
KIT D816	1.57 (0.80 - 3.08)	0.193
CSF3R	1.57 (0.56 - 4.42)	0.391
TET2	1.59 (0.73 - 3.45)	0.239
Cohesin complex	1.93 (1.01 - 3.69)	0.047
DHX15	2.70 (0.96 - 7.60)	0.060
Age >60 yrs.	3.23 (1.73 - 6.02)	<0.001
Non-primary AML	3.33 (1.51 - 7.32)	0.003

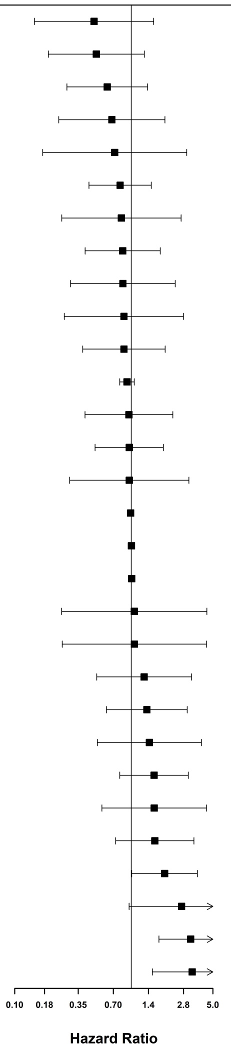

B

Variables	Hazard Ratio (CI 95%)	P-Value
del(Y) (males only)	0.39 (0.15 - 0.99)	0.047
FLT3-ITD	0.41 (0.10 - 1.69)	0.215
KRAS	0.56 (0.08 - 4.05)	0.562
del(Y)	0.61 (0.27 - 1.36)	0.225
del(7q)	0.61 (0.08 - 4.51)	0.630
NRAS	0.62 (0.24 - 1.57)	0.309
ASXL1	0.64 (0.23 - 1.80)	0.400
ASXL2	0.72 (0.35 - 1.48)	0.376
del(X) (females only)	0.73 (0.24 - 2.26)	0.586
del(X)	0.74 (0.28 - 1.92)	0.534
JAK3	0.80 (0.29 - 2.26)	0.679
ZBTB7A	0.85 (0.41 - 1.73)	0.649
del(9q)	0.86 (0.33 - 2.23)	0.751
CSF3R	0.91 (0.28 - 2.96)	0.879
FAT1	0.92 (0.28 - 2.99)	0.890
Hb (g/dL)	0.93 (0.81 - 1.07)	0.333
Cohesin complex	0.96 (0.44 - 2.07)	0.908
Platelets (10^9/L)	0.99 (0.98 - 1.00)	0.068
WBC (10^9/L)	1.00 (0.98 - 1.01)	0.730
Blasts in BM (%)	1.00 (0.99 - 1.02)	0.642
Female sex	1.03 (0.55 - 1.92)	0.930
FAT3	1.18 (0.28 - 4.90)	0.822
Clonal interference present	1.26 (0.62 - 2.58)	0.523
TET2	1.46 (0.65 - 3.29)	0.363
JAK2	1.72 (0.72 - 4.09)	0.222
Non-primary AML	1.86 (0.72 - 4.79)	0.200
FLT3-TKD	2.15 (0.76 - 6.06)	0.149
Age >60 yrs.	2.57 (1.36 - 4.85)	0.004
KIT D816	3.20 (1.68 - 6.08)	<0.001

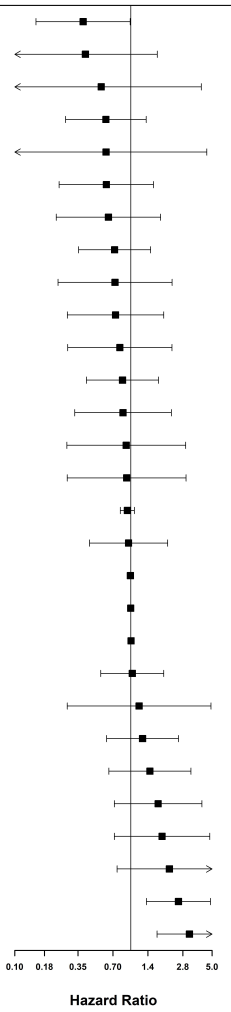

Supplementary Figure S7: Impact of *KIT* D816, *DHX15* and SMC1A mutations on outcome in *RUNX1/RUNX1T1* leukemia. Kaplan-Meier estimates indicate relapse free and overall survival for *KIT* D816, *DHX15* and SMC1A mutated patients with *RUNX1/RUNX1T1* leukemia. Number of patients in each group is shown in parentheses. P values were determined by log rank test.

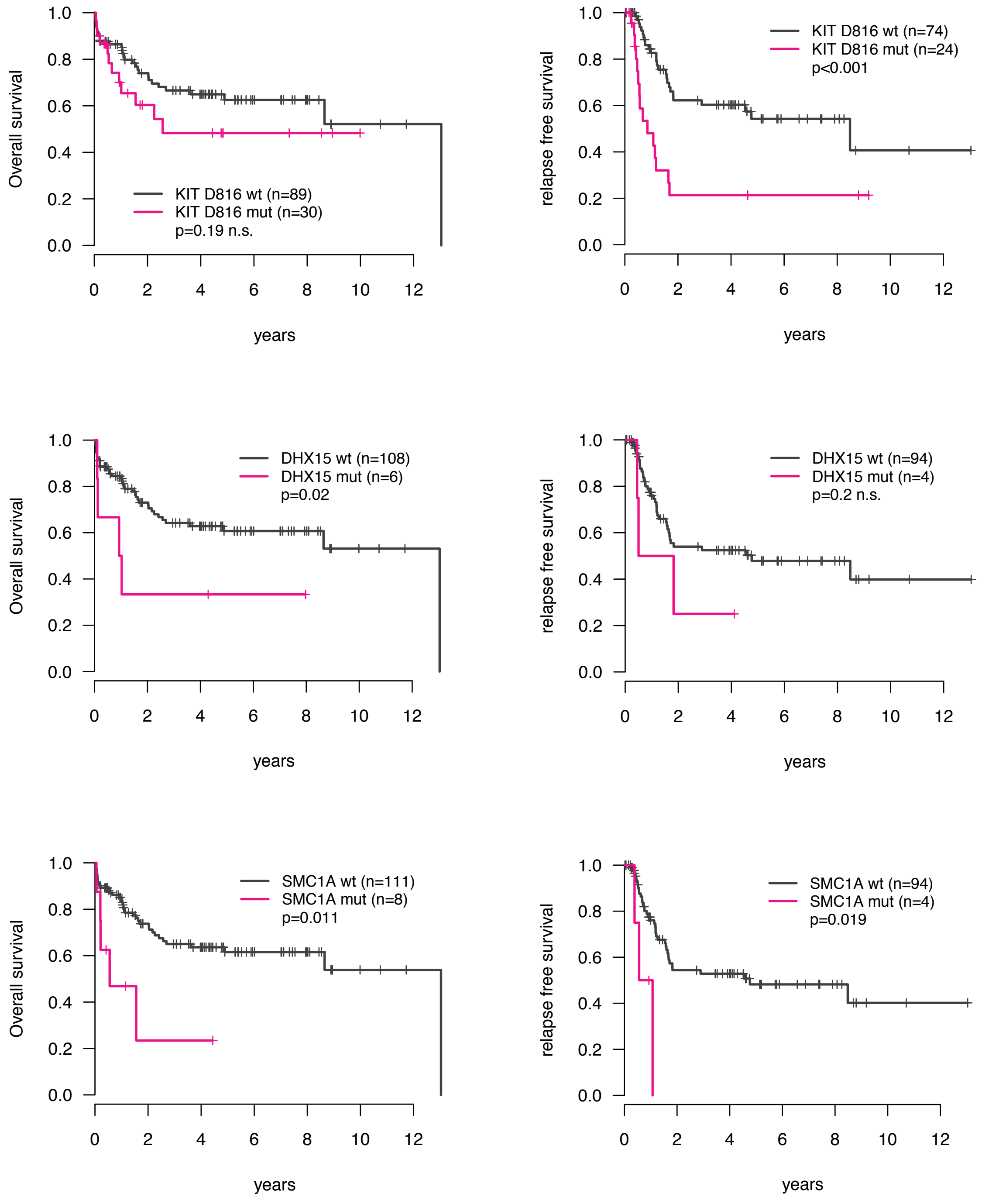

Supplementary Figure S8: Impact of the allelic burden of *KIT* D816 mutations on outcome in *RUNX1/RUNX1T1* leukemia. Kaplan-Meier estimates indicate relapse free and overall survival for *KIT* D816 mut^{high}, KIT D816 mut^{low} and wildtype patients with *RUNX1/RUNX1T1* leukemia. Mutant levels >25% were defined as *KIT* D816 mut^{high}. Number of patients in each group is shown in parentheses. P values were determined by log rank test.

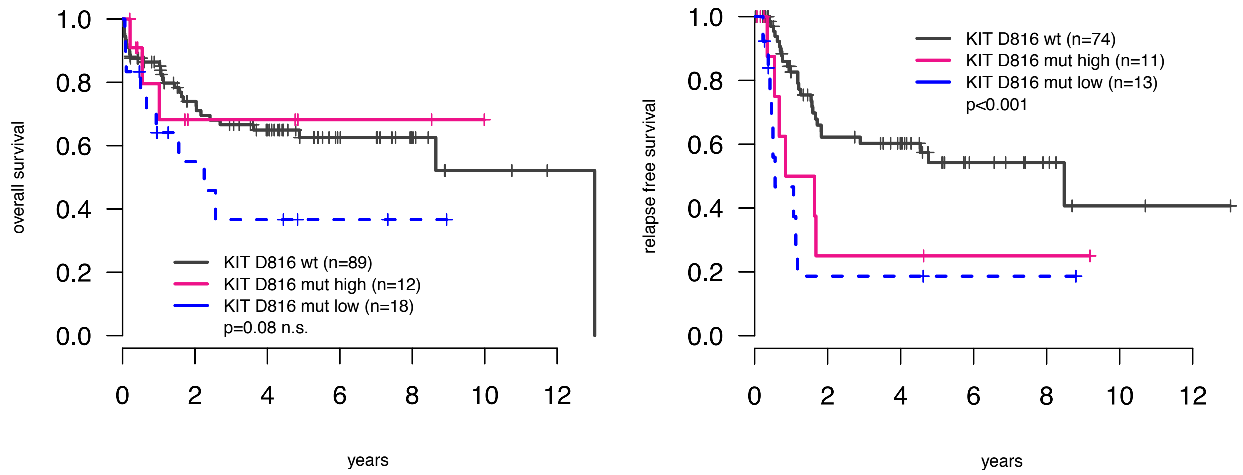

Supplementary Figure S9: Mutation profile of 10 CBF leukemia patients with SMC1A mutation. SMC1A mutations were found with high allelic burden (predominant clone). CBF leukemia subtype, gender and loss of sex chromosomes is shown.

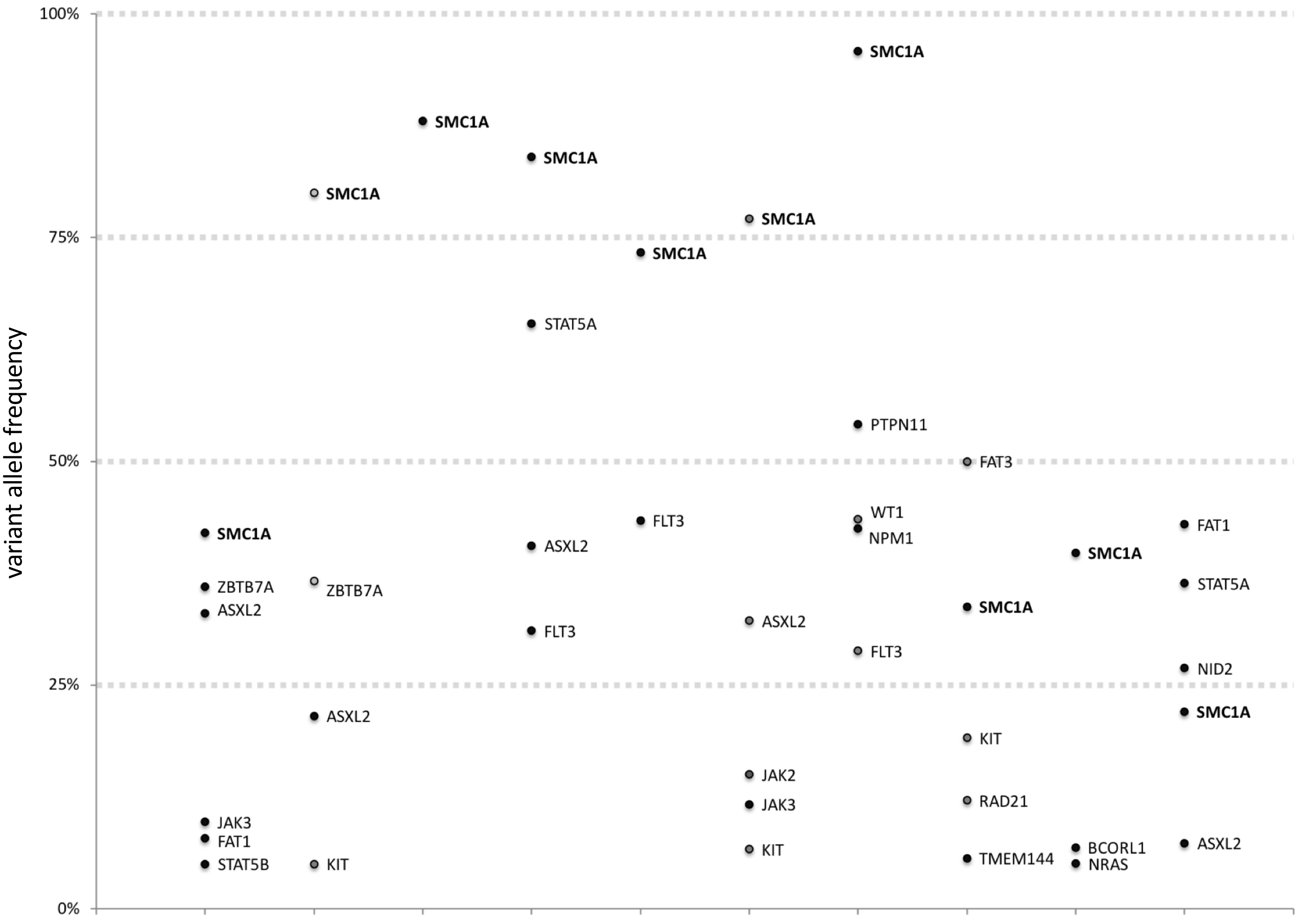

UPN	1	2	3	4	5	6	7	8	9	10
sex	XX	XY	XY	X,-X	X,-X	XY	XY	XX	XX	XY
subtype	t(8;21)	t(8;21)	t(8;21)	t(8;21)	t(8;21)	t(8;21)	inv(16)	t(8;21)	inv(16)	t(8;21)

Supplementary Figure S10: Impact of *TET2* mutations on outcome in *RUNX1/RUNX1T1* leukemia and correlation with age. A Kaplan-Meier estimates indicate overall survival and relapse free survival in RUNX1/RUNX1T1 rearranged leukemia for *TET2* mutated patients. Number of patients in each group is shown in parentheses. P values were determined by log rank test. **B** Boxplots of TET2 mutated patients (blue) and unmutated patients (orange) in comparison to age, each patient is representing one dot. Mean age of patients in each group is shown in parentheses. P values were determined by Welch two sample t-test.

A

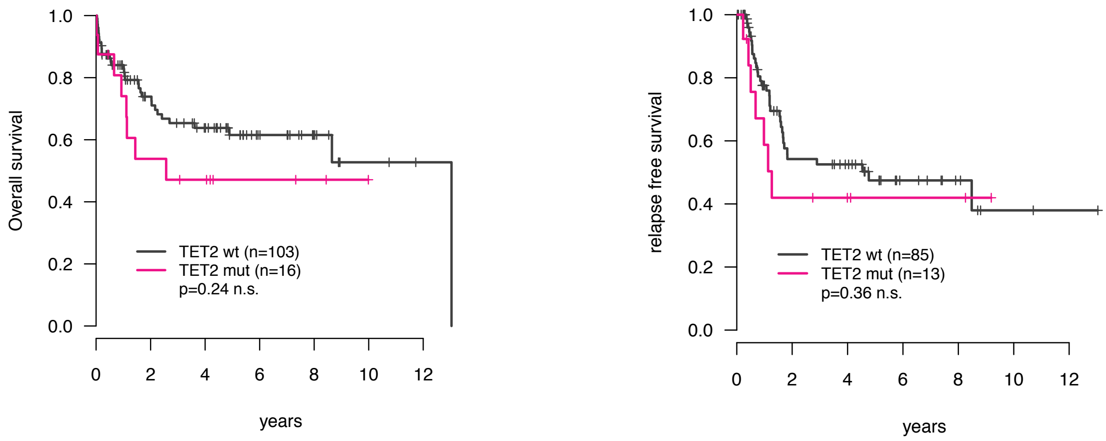

B

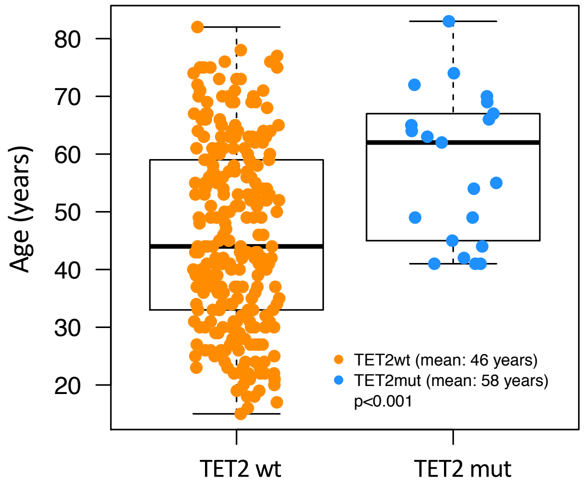

Supplementary Figure S11: Impact of *NFE2* and MN1 mutations on outcome in CBF leukemia. Kaplan-Meier estimates indicate overall survival and relapse free survival in CBF leukemia for *NFE2* and *MN1* mutated patients. Number of patients in each group is shown in parentheses. P values were determined by log rank test.

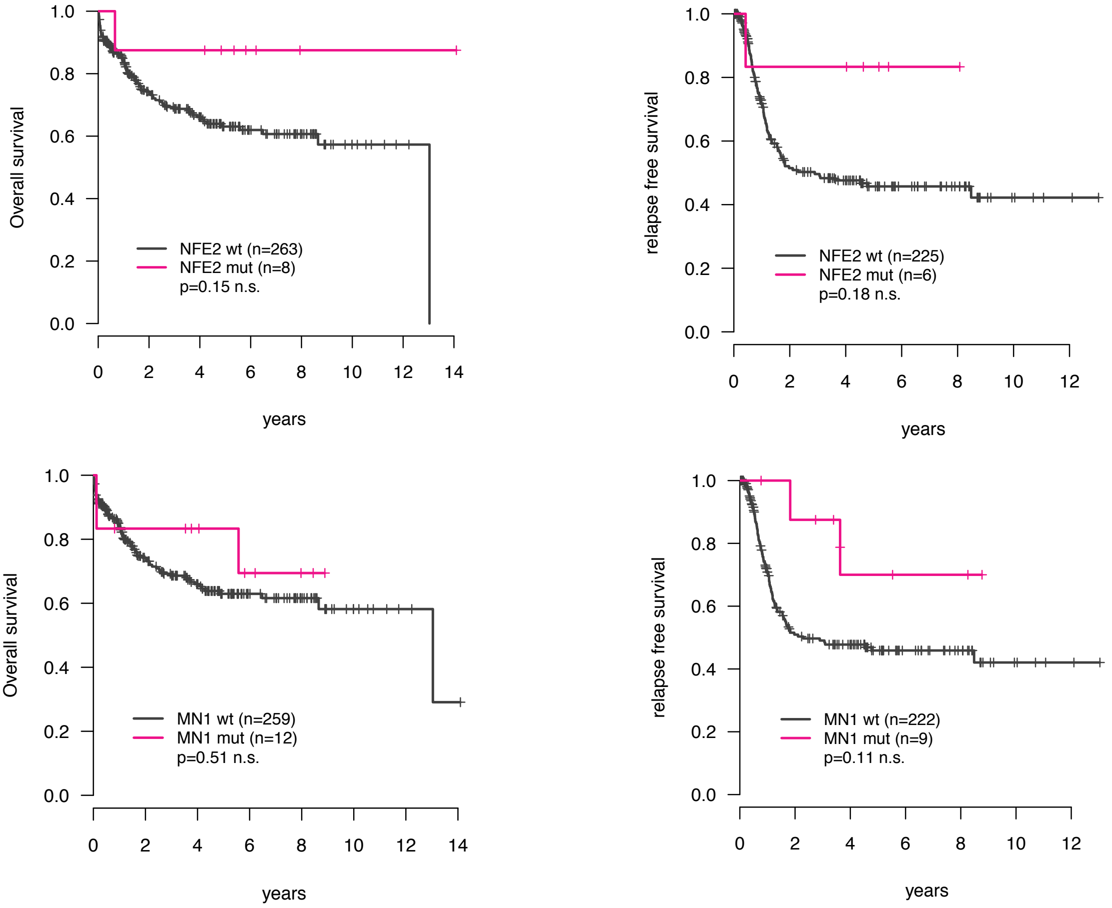

Supplement: Supplementary file 1 — Supplemental Information [file 41375_2019_697_MOESM1_ESM.pdf]
